# A guide to transient absorption spectroscopy for porous crystalline materials in photocatalysis

**DOI:** 10.1038/s41467-026-73511-4

**Published:** 2026-05-29

**Authors:** Alexander J. King, Liangji Chen, Andrew B. Maurer, Jier Huang

**Affiliations:** Department of Chemistry and Schiller Institute for Integrated Science and Society, Boston College, Chestnut Hill, Massachusetts, USA

**Keywords:** Porous materials, Excited states, Photocatalysis

## Abstract

Transient absorption spectroscopy has become an increasingly accessible method for probing the fundamental mechanisms of photocatalytic reaction and has provided important mechanistic insights across a wide range of systems. However, recent studies on metal-organic framework and covalent-organic framework based photocatalysts employ transient absorption primarily as a supplementary characterization technique rather than as a tool for gaining mechanistic insight. This perspective identifies recurring limitations in current practices and outlines strategies for more deliberate application of transient absorption spectroscopy in photocatalysis. By emphasizing clarity in data presentation and interpretation, we aim to elevate transient absorption spectroscopy from a routine characterization tool to a powerful approach for elucidating mechanistic photophysics of framework materials that govern their photocatalytic performance.

Two forefront classes of crystalline porous materials, namely metal-organic frameworks (MOFs) and covalent-organic frameworks (COFs), have successfully emerged over the past three decades and have rapidly become essential platforms for advancing research in environmental remediation and energy-related fields^1–3^. Their growing prominence is largely attributed to their exceptional structural features, such as periodic architectures, high surface area, tunable pore structures, and versatile chemical functionalities^1,2,4–6^, enabling broad applications in gas separation, catalysis, and energy storage and conversion^7–11^. In particular, their extended (often d-π/π-conjugated) networks facilitate efficient light harvesting and charge separation (CS), which positions them at the forefront of emerging photocatalytic materials^9,12–15^. Indeed, MOF- and COF-based materials already show promise across light-driven H₂ production via water splitting, CO₂ reduction, organic synthesis, H₂O₂ generation, and pollutant degradation^10,13,17^.

A central challenge in these photocatalytic reactions lies in the complexity of multielectron redox processes that often span timescales from sub-picoseconds to milliseconds or longer. Few techniques can resolve the transient intermediates involved across this broad temporal window, yet understanding their evolution is essential for uncovering the catalytic mechanisms to further accelerate the photocatalytic applications of MOFs and COFs^16^. Transient absorption (TA) spectroscopy, which is a powerful technique that can capture intermediate species with high temporal resolution, have demonstrated to be highly effective in elucidating the photophysical processes (e.g., excited state, charge transfer and separated state) that govern photocatalytic efficiency and define overall rate-limiting steps^17–19^. This capability extends to the field of MOF and COFs, as reflected by the growing number of studies utilizing TA spectroscopy to probe the excited state (ES) and CS dynamics, providing key insight into the photocatalytic mechanism under working conditions^17^.

Although these efforts have advanced the fundamental understanding of photophysical processes involved in photocatalysis, many recent works have yet to take full advantage of TA spectroscopy. Ultimately, limiting the ability to disentangle the rich ES evolution and CS dynamics inherent to these materials. In many recent studies that use TA spectroscopy, this technique is used primarily as a supplementary characterization tool that serves as either a check box to publish the paper or support the authors’ narrative rather than to investigate the material’s photophysical characteristics in a substantive way. In this comment, we highlight some recurring patterns we observed in recent literature that have limited the scientific impact of femtosecond (fs) and nanosecond (ns) TA studies on MOF and COF systems. Our aim is not to criticize the existing work, but to identify these recurring themes, offer improved strategies on data presentation and interpretation, and clarify how TA spectroscopy can be more effectively applied to these materials to advance the fundamental understanding of the photocatalytic mechanism.

## Working principles of transient absorption spectroscopy

TA spectra are differential absorbance measurements that track the sample’s absorption after excitation. The transient absorption signal, ΔA(λ, Δt), is measured as a function of probe wavelength (λ) and pump-probe delay time (Δt), resulting in a time-resolved spectral map. Here, ΔA is defined as the difference in probe absorption measured with and without photoexcitation from a pump pulse (Equation 1):

1 $$\Delta {{{\rm{A}}}}({{{\rm{\lambda }}}},\Delta {{{\rm{t}}}})={{{{\rm{A}}}}}_{{{{\rm{pump\; on}}}}}({{{\rm{\lambda }}}},\Delta {{{\rm{t}}}})-{{{{\rm{A}}}}}_{{{{\rm{pump\; off}}}}}({{{\rm{\lambda }}}},\Delta {{{\rm{t}}}}).$$

**Fig. 1:**
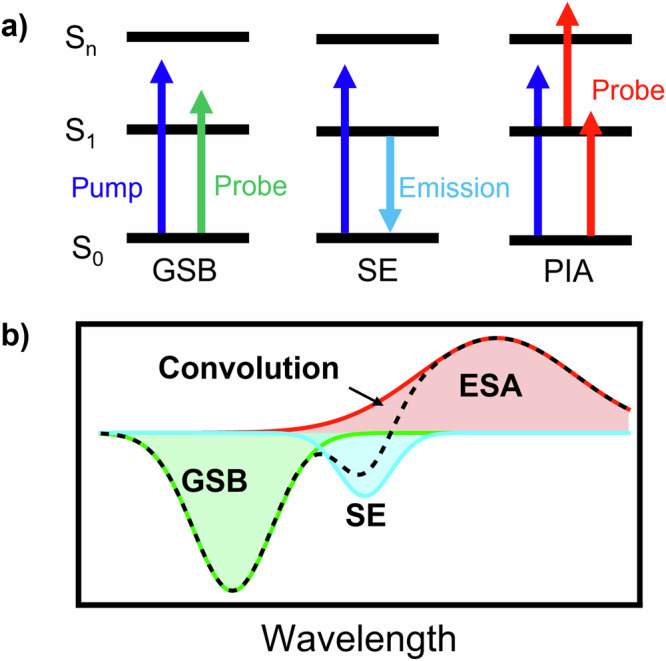
Fundamental transient absorption processes and their spectral signatures. **a** Schematic illustration of the three main transient absorption processes. The blue arrow corresponds to the pump excitation, the green arrow indicates the ground-state bleach (GSB) in the probe, red arrows represent photoinduced absorption (PIA), and the cyan arrow denotes stimulated emission (SE). **b** Simulated ΔA spectrum composed of three Gaussian features: GSB (green), ESA (red), and SE (cyan). The convolution of these signals yields the overall TA spectrum (black dashed line).

While the TA signal is defined by this relationship, experimental configurations can vary depending on the time delay (Δt) being probed. Ultrafast processes occurring on the femtosecond to picosecond timescale typically require short laser pulses and are commonly accessed using optical delay lines within the pump–probe beam path. In contrast, longer timescales in the nanosecond to microsecond regime are often measured using alternative approaches, such as electronically controlled probe delays or continuous-wave (CW) probe sources. Regardless of the temporal regime, TA spectra share several common spectral features that arise from transient species. In general, TA spectra typically comprise three primary contributions: ground-state bleach (GSB), photo-induced absorption (PIA), and stimulated emission (SE) (Fig. 1). Photoexcitation depletes the ground state and generates photoinduced products that give rise to these competing transitions. GSB and SE appear as negative signals (ΔA < 0), whereas PIA appears as a positive signal (ΔA > 0). Specifically in molecular frameworks, whether the electronic structure should be viewed as a collection of uncoupled or weakly coupled chromophores or as an extended organic semiconductor remains a debate in the community. As a result, the negative feature may represent a reduction in photogenerated carrier populations or exciton formation rather than solely ground-state depletion of molecular species^20–22^. The positive PIA typically arises from the absorption of photogenerated species formed after photoexcitation, including ES as well as reduced or oxidized states generated through charge transfer. Additional positive features may result from excitation density-dependent processes such as multi-exciton formation or exciton-exciton interactions. As illustrated in Fig. 1a, these processes can be visualized using a simplified energy-level diagram, and the measured spectrum (Fig. 1b) reflects the convolution of their overlapping spectral signatures. As these features can be challenging to assign, reporting steady-state UV-vis absorption and emission spectra provides important reference points for identifying GSB and SE signals and their evolution. In addition, spectroelectrochemistry can help identify absorption signatures of oxidized or reduced species, which may manifest as PIA features in TA spectra. Origins and the interpretation of the TA signal is provided wonderfully by Zhao et al.^18^.

## Common limitations in current transient absorption practices on MOFs/COFs

As described above, TA spectroscopy appears conceptually straightforward. However, translating this into practice requires careful consideration of pulse characteristics, precise alignment of delay stages, synchronized detection systems, and sample handling. Advances in ultrafast instruments have become increasingly accessible through commercial plug-and-play systems (e.g., Ultrafast Systems, Light Conversion, Clark-MXR Inc., Time Tech Spectra), reducing setup time and cost. While increased accessibility is undoubtedly a major strength, it also places greater responsibility on researchers to ensure careful experimental design, rigorous analysis, and transparent reporting. This responsibility becomes especially critical when studying structurally complex materials such as MOFs and COFs, even more so when attempting to correlate photophysical phenomena to photocatalytic activity (e.g., electron and hole transfer processes governing reactions such as H_2_O_2_ production, H_2_ evolution, or CO_2_ reduction). In these systems, structural inhomogeneity and strong scattering can substantially attenuate TA signals, hindering the acquisition of high-quality data and introducing optical artifacts that may be misinterpreted as genuine photophysical signal from the material. Such optical artifacts often arise from factors such as delay-stage misalignment, uncorrected group velocity dispersion, solvent contributions, or thin-film interference effects (e.g., Fabry-Pérot oscillations). Consequently, even with commercial plug-and-play systems require a deep understanding of the instrument to ensure reliable interpretation of the transient signals. In this section, we highlight recurring experimental and interpretative challenges and discuss how these practices can limit the reliability and clarity of photophysical conclusions used to interpret the behavior of MOFs and COFs photocatalysts.

A representative challenge in TA spectra of solid-state MOF/COF photocatalysts is the difficulty in disentangling true PIA dynamics from contributions arising from photoinduced refractive index changes. In the transmission mode measurements, refractive index changes can produce derivative-like features or long-lived responses that resemble PIA, thereby increasing the risk of mechanistic misassignment. This is also highlighted in a recent perspective by Pasanen el al.^23^. Transient responses in semiconductors and metallic films are often dominated by the photoinduced change in the refractive index rather than the “pure” absorption.

An additional consideration involves understanding instrumental limitations before drawing mechanistic conclusions from ultrafast measurements. For example, some reports claim relaxation lifetimes of a few hundred or even < 100 femtoseconds, which approach or fall below the time resolution of most commercial instruments. Even when an ultrafast laser’s fundamental pulse width is < 50 fs, nonlinear processes used to generate the pump and probe beams introduce temporal broadening, resulting in cross-correlation widths near 200 fs^19^. Without carefully accounting for instrument response, short-lived features may be misinterpreted as rapid CS, and failure to distinguish trapped carriers from reactive CS states can obscure whether long-lived species are capable of driving catalytic turnover or simply represent nonproductive recombination pathways.

Coherent artifacts at early times, particularly those occurring within the instrument response function, may also conflate early-time dynamics in transient spectra. Contributions from the solvent, cross-phase modulation, or thin-film interference effects can lead to misinterpretation of the data. While these effects are often difficult to eliminate, several steps can be taken to mitigate their influence. Measurements of the solvent system or substrate alone can help identify nonresonant responses; if performed under identical conditions as the sample, the resulting 2D surface can be subtracted from the sample data. In solution, keeping the pump-probe polarization angle at the magic angle suppresses anisotropic contributions, while power-dependent measurements can help distinguish nonlinear artifacts. In the event that these artifacts cannot be mitigated experimentally, researchers may choose to exclude the early time region when plotting or fitting kinetic traces. Considering these factors is essential when assigning dynamics on MOF/COF materials. Beyond these optical artifacts, the manner in which TA data are reported and visualized can strongly influences their interpretation. One common trend is the exclusive use of TA heat maps, often presented without supporting spectra or kinetic traces in the main text, with these critical components frequently relegated to the supporting information. Without accompanying spectra or kinetics, heat maps provide little more than a visual confirmation that the measurement was performed, which can reduce TA spectroscopy to a routine “check-box” measurement rather than a tool for mechanistic insight. Nevertheless, these plots still convey a general sense of spectral evolution and dynamics but offer limited quantitative information due to pixel-to-pixel interpolation that smooths noise and can mask subtle features in favor of a visually appealing image. Another issue when reporting only the heat map is the omission of pre-excitation (pre-t_0_) baseline data. Without early-time points, it is difficult to assess baseline stability, noise, or the origin of fast signal features, which can lead to misinterpretation of pump scatter or cross-phase modulation as meaningful photophysical events. Including pre-t_0_ data in either wavelength-dependent kinetic traces or time-dependent spectra (e.g., waterfall plots) is therefore essential for evaluating the reliability of transient signals.

Limitations also arise in the reporting and analysis of kinetic traces. In certain studies, transient kinetics are presented in a simplified manner, with either the full spectral dynamics represented by a single wavelength or multiple lifetimes condensed into one average or effective value. In systems as complex as MOFs and COFs, selecting a single wavelength to represent ES dynamics can overlook processes such as CS and charge trapping, exciton delocalization or localization, and energy transfer; while this approach may be appropriate when a single feature dominates and relaxes uniformly, it is rarely justified or contextualized in the text. Similarly, multi-exponential fits are sometimes applied without justification of their physical basis. Even when multiple components are justified by rate-law behavior, the resulting lifetimes are presented without further interpretation. While condensing multi-component dynamics into average- or half-lifetimes may facilitate comparison across materials, it can also obscure mechanistic details, and authors should exercise caution when reporting an average ES lifetime^24^. For guidance on proper data processing, analysis, and modeling, we encourage readers to consult a 2012 review by Sliwa et al.^25^.

TA spectra are inherently multicomponent, with convoluted spectral and kinetic contributions that are not readily resolved by single-wavelength analysis or averaged lifetimes. Methods such as singular value decomposition (SVD) and global analysis provide a means to separate these contributions and identify the underlying photophysical processes, such that researchers can connect the photophysics to photocatalytic processes. SVD is a mathematical technique that decomposes a dataset into its orthogonal spectral and temporal components, weighted by their contribution to the overall signal. In TA, SVD is commonly used to determine the number of significant components and to distinguish signal from noise^26,27^. While SVD provides insight into the number of significant components present in a dataset, it does not directly assign physical meaning to these contributions. To extract mechanistic information, global analysis approaches such as decay-associated difference spectra (DADS) and evolution-associated difference spectra (EADS) are employed, where kinetic models are applied in a simultaneous fit across the full dataset. In DADS, each lifetime is associated with a distinct spectral component, which may correspond to a specific species. In contrast, EADS assumes a sequential rate model, yielding spectra that represent the apparent time evolution of intermediates along a defined pathway. The application of these methods is often included in commercial software, but also can be accomplished through open-source options, including KiMoPack^28^, OPTIMUS^29^, and Glotaran^30^ most of which include or have tutorials for use available. More details of these analytic methods can be found in the works of Slavlov^29^ and van Grondelle^31^.

## Recommendations for effective data representation

In this section, we offer recommendations for improving the clarity and interpretability of TA data presentation. To give readers a clear understanding of the data being presented, we encourage authors to list relevant sample conditions in the figure caption, such as sample phase, solvent if appropriate, pump wavelength, and probe region. For example, “Panel b corresponds to transient absorption data collected on the thin film COF using a 355 nm pump and a probe white light continuum generated with a sapphire crystal”. Visualization of TA data can directly influence how features of perceived and interpreted by the authors and the readers. Our aim is to encourage better practices that support meaningful insight into ES dynamics. Examples of suboptimal visualization appear frequently in the literature, however, to clearly demonstrate best-practice approaches without referencing specific studies, we simulated a TA data set that includes representative features of an excited state absorption (ESA), GSB, and the growth of a low-amplitude GSB in order to illustrate how visualization choices influence the interpretation of transient features and their relevance to photocatalytic function.

**Fig. 2:**
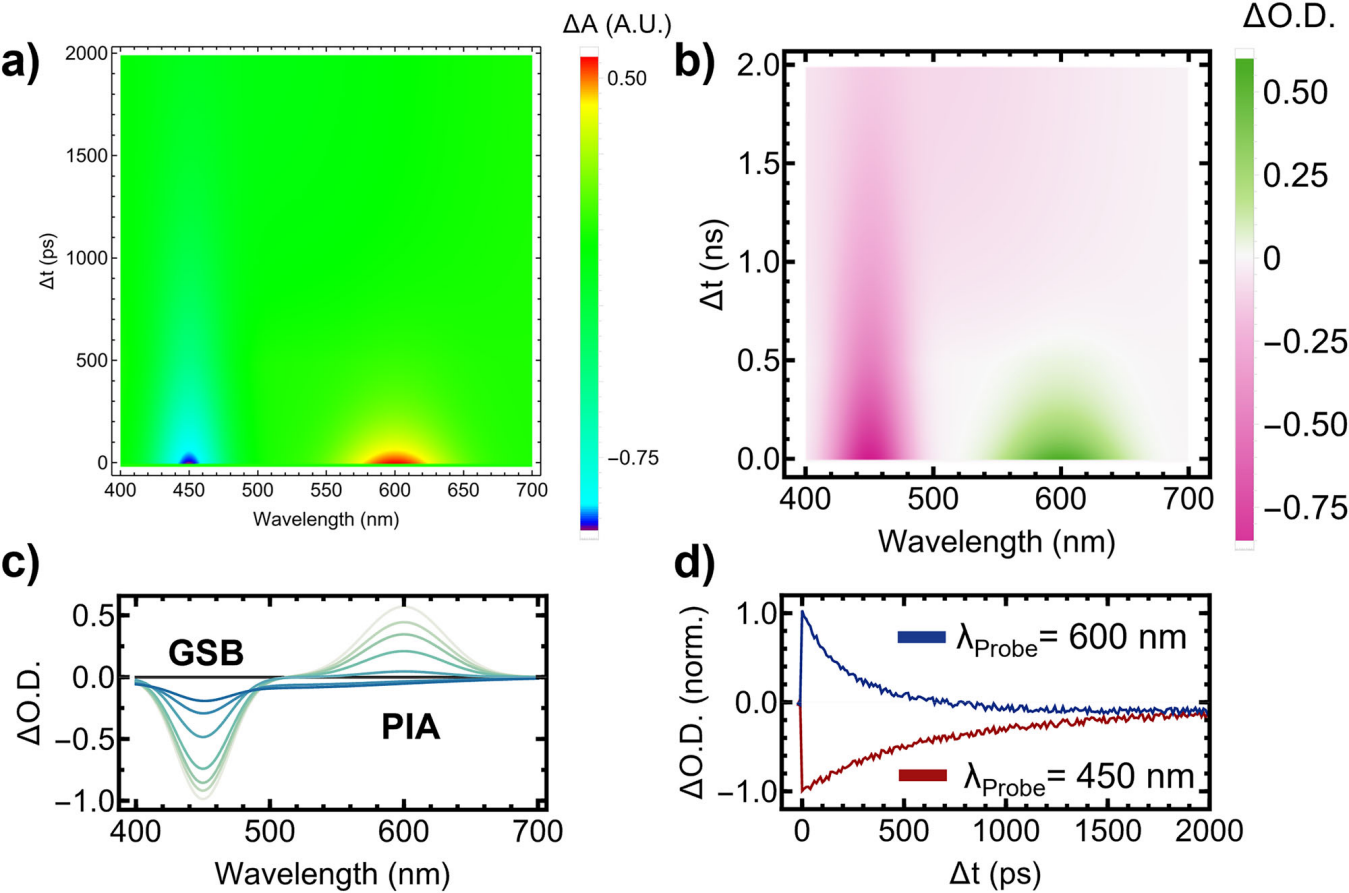
Simulated transient absorption data comparing conventional visualization with improved representation. **a** Corresponds to typical TA data commonly found in literature. In this representation data set, the background is dominated by green tones and hides a subtle feature, axes are difficult to read, and the use of the rainbow color scheme can limit accessibility for readers with color vision deficiencies. In addition, the color scale is sparsely labeled, making this panel useful for qualitative information. **b** Corresponds to the same dataset with improved representation. Here, the background corresponds to white tones, while the minimum and maximum signal amplitudes (ΔO.D., change in optical density) consist of contrasting colors. In addition, pre-t_0_ data highlights no lingering singles, the scalebar as multiple values. In some cases, the transient signal does not solely reflect changes in material absorption; in such instances, reporting the signal as optical density may be more appropriate. **c** Corresponds to a representative time evolved spectrum set, highlighting the data displayed in panels a and b; the inset displays the selected time delays for each spectrum. The ground state bleach (GSB), photoinduced absorption (PIA), and time delays indicate are clearly labeled. **d** Corresponds to the kinetics for the negative and positive features and clearly indicates the corresponding features the kinetics are plotted from.

One very common example of poorly represented data in the literature is represented in Fig. 2a, where the heatmap appears to be mostly a uniform green background and only the most intense features are distinguishable. The low-intensity feature in the data that could be representative of a new ES species is clearly masked by the color scaling in this example. The corresponding scale bar is also sparsely labeled with only two values, making the signal amplitude challenging to ascertain. In contrast, panel b applies a more appropriate color scaling that allows individuals with color vision deficiencies to clearly interpret the data. The low intensity feature that forms at later times becomes more evident to the reader by the diffuse purple feature. Panel b highlights that diverging colormaps are not always necessary to convey temporal and spectral structure effectively. This example illustrates how visualization choices can obscure subtle but potentially mechanistically relevant spectral features. Adjusting color scaling or simplifying colormaps improves visibility without altering the underlying data.

Heatmaps offer a convenient way to display the full TA dataset, but they are often less effective at conveying subtle spectral and temporal details. Panel c shows selected time-resolved spectra, making it easier to track the evolution of both GSB and ESA features over time. This view helps reveal spectral shifts, broadening, and the appearance or decay of features that are often obscured color maps, especially when an inappropriate color gradient is used. Panel d displays the corresponding kinetic traces at selected wavelengths, normalized to enable direct comparison of their temporal behavior. In this example, we retain the sign of the signal to preserve the distinction between GSB and PIA. These kinetic traces directly convey the distinct lifetimes and temporal evolution of different excited states, which are critical for interpreting the underlying photophysics. Although heatmaps provide an overview, the combination of selected spectra and kinetic traces more effectively supports mechanistic interpretation.

Even high-quality TA data can fall short if it is not presented in a way that supports mechanistic interpretation. Simple choices such as selecting an appropriate color gradient, including baseline data, showing spectra at meaningful time delays, and plotting normalized kinetics in either linear or log time can dramatically improve clarity and interpretability. While we have only provided suggestions on how to represent data, our goal is to encourage more deliberate and transparent presentation practices. By doing so, researchers can more effectively communicate ES behavior and enable more meaningful comparisons across MOF and COF systems.

## Practical considerations for interpreting TA measurements

Interpreting TA data in MOF and COF materials requires a comprehensive analysis of the spectral evolution of all transient features (e.g., GSB, PIA, SE). Such analysis provides critical mechanistic insight into the identity of the intermediate species formed during photocatalytic reactions and clarifies the associated reaction kinetics. Rather than relying on lifetimes alone, interpretation should focus on how specific spectral signatures reflect energetic processes and how those features evolve over time. The following discussion presents a practical approach to TA interpretation in MOFs and COFs photocatalysts, with emphasis on connecting spectral features and their temporal evolution to the underlying photophysical processes and material structure, supported by representative examples.

It is important to report the physical phase of the material used for TA measurements (e.g., suspension or solid-state thin film). While suspensions can often be advantageous because solid-state artifacts such as thin-film interference are minimized, framework materials are frequently difficult to disperse sufficiently such that the particle size does not obstruct the beam or produce significant scattering. Researchers should also consider whether the dielectric response of the solvent contributes to the measured signal. For solid samples, films should be sufficiently transparent and smooth to minimize scattering effects. In solid-state measurements, the relative orientation of the pump and probe beams can selectively probe changes in the transition dipole moment, meaning that the measured signal may depend on sample orientation or polarization conditions. In addition, solid samples may be more susceptible to laser-induced damage or thermal accumulation since there is no solvent to dissipate heat from the excited region, requiring careful control of pump fluence and repetition rate

As discussed in the working principles of transient absorption spectroscopy, a typical TA spectrum of MOFs/COFs consists of both negative and positive transient features. Negative signals are typically attributed to a GSB and/or SE, although a SE feature in solid-state or framework systems is rare due to many nonradiative recombination pathways^32–34^. The spectral shape of the GSB often mirrors the steady-state absorption, although deviations may arise from selective bleaching or redistribution of oscillator strength^35,36^. Narrow bleach features often suggest localized transitions, whereas broadened or featureless signals may reflect exciton delocalization or structural disorder^34,37,38^. Structured bleach, such as vibronic shoulders, implies well-defined transitions, while temporal shifting or flattening may indicate carrier migration, spectral diffusion, or the emergence of new species^36,39^. These nuances are especially pronounced in frameworks with strong coupling or heterogeneous environments, where the bleach feature is not a simple decay. In addition to negative signals, positive transient features require equally careful interpretation. The positive PIA features can arise when photoinduced products (e.g., ES, oxidized or reduced state) absorb additional photons to reach higher-lying states but may also originate from photoinduced changes in the refractive index^23,40^, surface plasmons^41^, or Stark effect^42^. In MOFs and COFs materials, these PIA signatures often reflect transitions from bound excitons^43^, charge-transfer states^44^, with their spectral shape/position providing insight into the identity and dynamics of these species.

Not all relevant photocatalytic intermediates result in an observable optical transition, making identification challenging using TA spectroscopy alone. Complementary techniques such as electron paramagnetic resonance^45^ (EPR) can identify paramagnetic radicals, and time-resolved X-ray absorption spectroscopy^46^ (TR-XAS) can identify changes in a metal’s coordination environment. A simpler approach involves performing additional TA measurements under catalytic conditions (e.g., in the presence of electron or hole scavengers) to isolate specific charge-transfer pathways.

After establishing a mechanistic understanding of these transient species through careful analysis of their spectral characteristics and temporal evolution, quantitative kinetic analysis should follow, as it can yield critical insights into catalytic reaction dynamics, CS efficiency, and the identification of the rate-determining step. For example, a rapidly decaying bleach typically reflects ground state recovery via recombination or nonradiative relaxation, whereas a persistent bleach may indicate trapped carriers or spatially separated charges due to the slow repopulation of the ground state^35,47^. In addition, the persistence of a spectral feature, regardless of signal sign, should be carefully evaluated in terms of whether the associated state can and does participate in redox chemistry in photocatalysis. Long-lived states are often assumed to correlate with improved catalytic activity; however, without establishing whether these states are energetically aligned and spatially accessible to either reactants, such as CO_2_ or protons or free carriers, lifetime alone cannot serve as a means for justification in catalytic performance. TA interpretation should therefore consider not only temporal evolution but also whether the observed species plausibly participates in the catalytic cycle.

Given the complexity of photophysical processes underlying MOF and COF photocatalysis, a systematic interpretive strategy is essential for relating transient signatures to catalytic function. To avoid treating TA spectroscopy as a routine characterization step, we propose a structured approach for disentangling complex spectra and identifying photoexcited intermediate states that are relevant to photocatalytic mechanisms. An effective strategy begins with elucidating the ES dynamics of the corresponding molecular building blocks (e.g., organic linkers) and/or a molecular model (i.e., a single strut in MOFs/COFs), provided that synthesis of the model complex is feasible. This involves establishing the spectral positions of GSB, SE, and ESA features, along with their lifetimes and temporal evolution. Doing so helps identify transient signals that are intrinsic to the molecular unit and provides a reference point for interpreting the more complex spectra of the extended framework. We note that care must be taken to ensure the precursor remains monomeric, as aggregation can introduce new spectral features and excited-state dynamics that differ from those of the isolated chromophore. Interestingly, molecular aggregation studies may still provide insight into the extent of electronic coupling within the framework. In addition, solvent polarity and dielectric environment may influence the resulting spectra, and these factors should be considered when comparing monomer measurements to framework materials.

Upon formation of MOFs or COFs, in most cases, the extended framework no longer behaves as a discrete molecular species. Instead, additional processes such as charge transfer or delocalization may be introduced and give rise to new transient signatures^48^. One method to assess these additional effects is to compare the vibronic structure and bandwidth of the GSB in the molecular model with that in the extended framework. Narrowing with increased vibronic resolution may indicate localization on a single chromophore, whereas broadening or loss of vibronic structure suggests delocalization across multiple units, as we have observed in our previous studies^34,49^. ES lifetimes can differ substantially between an extended framework and its corresponding model complex, reflecting the introduction of additional pathways such as CS, charge transport, and trapping that are not accessible in isolated molecular units. Increased electronic connectivity can, in certain systems, reduce geminate recombination and prolong the lifetime of long-lived species. For example, we have observed a substantially longer ES lifetime in a zeolitic imidazolate framework (2.9 μs) compared to its single-unit strut (3.7 ps)^50,51^. These examples illustrate how comparisons between molecular models and their extended frameworks can disentangle overlapping signals, assign the origins of key spectral features, and relate them to underlying photophysical processes relevant to photocatalytic function.

A complementary strategy involves comparing frameworks with and without a catalytic center to identify and quantify CS dynamics and directly correlate them to photocatalysis. In one of our previous studies^52^, we incorporated a Co(bpy)Cl_2_ catalytic center into a Ru(bpy)_3_-functionalized UiO-67 framework and directly compared their TA responses. While the Ru-only framework exhibited ES decay consistent with intrinsic recombination, incorporation of the Co moiety led to a pronounced acceleration of the Ru ESA decay. This reduction in ESA lifetime was assigned to ultrafast electron transfer from the Ru photosensitizer to the Co catalytic center, establishing CS as the initiating step for hydrogen evolution. In contrast, in our studies of Re-functionalized COFs^53^, CS did not manifest solely as a reduction in lifetime but instead through the emergence of a new long-lived feature upon incorporation of the Re center. In the unmetallated framework, the intrinsic ESA decayed completely within 500 ps, whereas the introduction of the Re site generated a new species persisting well beyond 14 μs, which we assigned to having ^3^MLCT character. As a result of this long-lived intermediate state, we concluded that the photocatalytic CO_2_ reduction to form CO becomes observable with Re-f-COF, whereas the unmetallated framework exhibits no detectable activity. In both examples, direct comparison of frameworks with and without catalytic centers helps identify CS states that are responsible for enhanced catalytic turnover. The use of electron, hole, or triplet scavengers can further support these assignments by selectively perturbing charge-transfer pathways. For example, the loss of a long-lived feature in the presence of a hole scavenger suggests involvement of a hole or CT state, while electron scavengers probe electron involved dynamics, and molecular oxygen can be used to probe triplet-states. Common electron scavengers include methyl viologen, silver nitrate, and benzoquinone, whereas methanol, ethanol, and triethanolamine (TEOA) are frequently used as hole scavengers. These examples demonstrate that careful experimental design enables TA to provide mechanistic insight directly linked to photocatalytic performance.

## Summary

MOFs and COFs have quickly emerged as promising materials for photocatalysis, owing to their extended networks that generate and separate excitons with remarkable efficiency. TA spectroscopy offers a unique opportunity to connect structural design with catalytic function, yet in practice, it is often treated as a routine “checkbox” technique. As a result, many studies lack substantial interpretation. Meaningful analysis requires careful attention to time-evolved spectral features, recognition of experimental artifacts, transparent data representation, and rigorous evaluation of ES dynamics. Strategies such as incorporating baseline stability, presenting spectra alongside kinetics, using appropriate color scaling, retaining signal sign to distinguish GSB from ESA, and comparing molecular precursors/models with extended frameworks can help disentangle overlapping signals and clarify intermediate species, including CS, exciton migration, and trap-mediated recombination. Coupling these practices with careful attention to experimental artifacts and transparent reporting ensures that TA spectroscopy is applied not as a routine measurement, but as a deliberate tool for mechanistic insight. To provide researchers with a structured reference, we summarize common pitfalls and recommended practices in Table 1. As the photophysics of these frameworks continue to evolve, rigorous use of TA will be essential for translating complex signals into clear narratives that guide the rational design of next-generation photocatalytic materials.

**Table 1:** Common Pitfalls and Recommended Best Practices in TA Analysis

Pitfall	Best Practice	Impact
Reporting lifetimes comparable to or below the instrument cross-correlation without validation	Compare extracted kinetics to the instrument response function and report cross-correlation width	Prevents overinterpretation of ultrafast charge separation or recombination
Assigning positive or derivative-like features solely to ESA	Evaluate possible refractive index contributions and verify spectral shape and fluence dependence	Reduces mis-assignment of transient species
Poor heatmap color scaling that obscures low-intensity features	Use appropriate color gradients, clearly labeled scale bars, and supplement with spectra and kinetics	Improves visibility of subtle dynamics
Representing full spectral dynamics using a single probe wavelength	Present time-resolved spectra and normalized kinetics at multiple wavelengths	Preserves spectral context and avoids oversimplified interpretations
Collapsing multi-exponential fits into a single average lifetime	Interpret individual kinetic components and discuss their physical basis	Prevents masking of competing excited-state pathways
Oversimplification of multicomponent data	Use SVD to determine the number of significant components and apply global analysis with physically justified kinetic models	Resolves multicomponent dynamics
Assuming long-lived signals directly imply improved catalytic performance	Evaluate energetic alignment and accessibility to reactants (e.g., CO₂, protons)	Avoids misleading structure-function correlations
Interpreting framework spectra without molecular reference systems	First, characterize molecular models to define intrinsic transient signals	Distinguishes chromophore behavior from framework-induced processes
Ignoring changes in bleach morphology or vibronic structure	Compare bandwidth and vibronic resolution between molecular and extended systems	Reveals localization, delocalization, and electronic coupling effects
Assigning catalytic enhancement based solely on lifetime changes without appropriate structural controls	Acceleration of excited-state decay or emergence of new long-lived metal-associated absorption features	Direct identification of charge-separated states responsible for catalytic turnover
